# Antibiotic resistance and R&D failure: The need for near real-time disaster research

**DOI:** 10.4102/jamba.v12i1.795

**Published:** 2020-03-12

**Authors:** Chris W. Callaghan, Oren Dayan

**Affiliations:** 1 School of Economic and Business Sciences, University of the Witwatersrand, Johannesburg, South Africa; 2 Milpark Business School, Milpark Education, Johannesburg, South Africa

**Keywords:** disaster, disaster risk research, antibiotic resistance, innovation, R&D, probabilistic innovation theory

## Abstract

Increasing antibiotic resistance across the world seems to reflect a failure of research and development (R&D) to keep pace with societally important disaster risks. This article uses the example of steadily increasing antibiotic resistance to question whether current R&D systems are able to timeously deal with certain societally important research problems. A review and discussion of new theoretical developments is offered, to suggest how novel technologies might be applied to improve the efficiency and effectiveness of health-related disaster risk research. This article seeks to make a conceptual contribution through a critical review and synthesis of novel theory. Theoretical propositions are derived from conceptual analysis. Four key challenges are related to the derived propositions, to derive guidelines for how the disaster risk management process can be supplemented to improve its near real-time research capability. The theoretical propositions derived here relate to certain overarching challenges facing disaster risk research. The theoretical arguments made in this article seek to offer a heuristic perspective that may be useful to those seeking to apply novel technologies in disaster risk research to address societally important research problems such as antibiotic resistance. This research identifies evidence of the failure of the contemporary research system to solve problems like antibiotic resistance. On the basis of a synthesis of novel literature and theory, conclusions suggest certain useful avenues for the improvement of the research process. Urgency is recommended because of mounting societal costs of slow research responses to societal problems.

## Introduction

The pace at which antibiotic resistance is spreading seems to be faster than the pace at which we can develop new drugs to halt its progress (Huttner et al. 2013). This problem is exacerbated as certain pharmaceutical firms have closed down their antibiotic resistance research and development (R&D) units because they are not as profitable as other domains (Harbarth, Theuretzbacher & Hackett 2015). Microbes are becoming resistant to antibiotics – a ‘once off’ treatment, encouraging pharmaceutical firms to develop other drugs that offer a longer stream of income, such as more profitable medicines for chronic conditions that have to be taken for longer, or even for life.

The emergence of antibiotic resistance to tuberculosis (TB) offers an example of this trend. In the 1990s, multidrug-resistant (MDR) TB emerged worldwide, coming to be defined as resistance to isoniazid and rifampin. It requires less effective and more costly second-line drugs (Centers for Disease Control and Prevention [CDC] 2006). In early 2005, in rural KwaZulu-Natal, a province of South Africa, cases of MDR-TB were accompanied by extensively drug-resistant (XDR) TB – a strain also resistant to capreomycin, kanamycin and amikacin (Raviglione & Smith 2007).

Since then, a TB strain has been described as ‘totally’ drug-resistant (TDR-TB) (Klopper et al. 2013), or as virtually untreatable TB. It was initially identified in India, Iran, Italy and South Africa (Slomski 2013). The World Health Organization does not endorse the TDR classification, appearing to be more optimistic about its management; nevertheless, this strain is eventually expected to emerge wherever MDR-TB and XDR-TB are prevalent. According to Koul et al. (2011:483/4), TB is now more prevalent across the world than it has ever been in history, yet our approaches to developing new TB drugs are too slow and problematic – the decades old treatment options we have are dwindling in efficacy.

Koul et al. (2011:484) stressed that increased resistance to antibiotics raises the possibility of a return to a pre-antibiotic TB era. Tuberculosis is just one dimension of what seems to be a growing problem of antibiotic resistance. The increasing disaster potential of antibiotic resistance is highlighted by Nathan (2004:899) in his question, posed in the journal Nature – the ‘challenge is this: what can be done about the level of antibiotic R&D, which has long been insufficient to meet the needs of most populations, and now is plummeting?’ Given the disaster threat of antibiotic resistance, the objective of this article was to provide a conceptual review of what seems to be a growing knowledge problem in biomedicine, namely, the threat of a return to a pre-antibiotic era – a research problem that exists within a global R&D system. In doing so, we seek to offer a predictive model in the form of theoretical propositions for how novel theory and developments in technology might ultimately address the problem of slow disaster research response to potentially catastrophic threats.

This article seeks to contribute to disaster risk theory literature in the following ways. Given the mounting evidence of a global slowdown in returns to research (Cowen 2011; Gordon 2016), and theoretical models that explain why it is occurring (Kortum 1997; Segerstrom 1997), we connect this literature to evidence of growing threats such as TDR-TB as well as long-standing evidence of spreading antibiotic resistance (Goossens et al. 2005; Kumarasamy et al. 2010; Neu 1992). In doing so, this synthesis is used to argue that a failure to prioritise the discovery of novel theories and new technologies and to apply these *to the improvement of the discovery process itself* could have catastrophic consequences. This article therefore reviews different literatures in light of alternative bodies of theory that *demonstrate how a global slowdown in innovation might be reversed* through different mechanisms. Examples of theory that predict these mechanisms include theoretical predictions associated with the interactive contribution of novel technologies of the fourth industrial revolution (Schwab 2017), or the second machine age (Brynjolfsson & McAfee 2014), which suggests how technologies can leverage or amplify human collective intelligence (and therefore disaster research problem solving). Having foregrounded certain of the ideas we synthesise in this article, we now revisit literature relating to the threat of growing microbial resistance to drugs.

## Growing resistance to all forms of antibiotics

How does bacterial resistance to antimicrobial agents occur? Bacterial resistance to antimicrobial agents occurs through different mechanisms. One mechanism acts through chromosomal changes and the way bacteria can exchange between themselves genetic material via plasmids and transposons (Neu 1992). Bacteria can therefore exchange genetic material amongst themselves, which makes them resistant to antibiotics.

As bacteria can share antibiotic genes between them, it is difficult to stop their spread. Mobile genes on plasmids can spread though bacterial populations, enabled by ‘unprecedented air travel and migration’, which allows bacterial plasmids and clones to be transferred across continents (Kumarasamy et al. 2010:597). This is particularly problematic, as one region might use antibiotics conservatively, but resistance can now spread across regions. As an example of this growing threat, Kumarasamy et al. (2010) identified isolates that show the spread of MDR Enterobacteriaceae between the United Kingdom, India and Pakistan.

According to Huttner et al. (2013:1), microbes ‘have globalized along with their hosts, while at the same time antimicrobial consumption by these hosts – both humans and animals – has exploded’, and the ‘gene pool for antimicrobial resistance has never been so accessible, nor its selection pressure so strong’. Harbarth et al. (2015) argued that a ‘discovery void’ has developed over the past 20 years in the form of a failure to respond to this threat, as most pharmaceutical companies have closed down their antibiotic resistance R&D units.

If most pharmaceutical companies have closed down their antibiotic R&D units, this discovery void might contribute to the return to a pre-antibiotic era. Given the incentive structures of the global pharmaceutical industry, it must be asked: is antibiotic resistance inevitable under the current global R&D system? According to Huttner et al. (2013:1), humans are ‘being outrun’, as antibiotic resistance is overtaking our R&D capabilities. According to Huttner et al. (2013):

[*H*]umans are being outrun: there have been no successful discoveries of new classes of antibiotics since 1987, while new, multiresistant pathogens such as carbapenem-resistant enterobacteriacae (CRE) are spreading with unprecedented alacrity. (p. 1)

In an age of multi-drug resistance, new approaches such as the use of bacterial viruses (phages) to infect bacteria at the site of infection are receiving increased attention (Lin, Koskella & Lin 2017). A review of medicinal chemistry tools and challenges currently faced by antibiotic research is provided by Singh, Young and Silver (2017). It is not clear, however, if such approaches will ultimately be successful.

### The consequences of antibiotic resistance for poor populations

Data suggest that ‘antimicrobial misuse, prophylactic use, diagnostic imprecision, and interpersonal spread are key factors in the selection and dissemination of resistant strains’, whereby all ‘these factors are promoted by poverty at the individual patient, health system, and national levels’ (Okeke 2009).

Antibiotic consumption seems to have increased the most in developing countries. Over the period 2000–2010, global consumption of antibiotic drugs increased by 36%, of which Brazil, Russia, India, China and South Africa (BRICS countries) were responsible for 76% of the increase (Van Broeckel et al. 2014).

To prevent ‘a striking rise in resistance in low-income and middle-income countries with large populations and to preserve antibiotic efficacy worldwide’, coordinated efforts are needed by the international community to promote more conservative use of antibiotics (Van Broeckel et al. 2014:1). However, as mentioned previously, if a microbe develops resistance in one region, it can quickly spread to all other regions of the world; hence, supplementary strategies are needed.

### Co-infections of human immunodeficiency virus or tuberculosis and human immunodeficiency virus or malaria

Antibiotic resistance is accelerating because of co-infections, such as those between human immunodeficiency virus (HIV) and TB, which exacerbates the morbidity and mortality of each alone (Deidrich & Flynn 2011). Over a billion people are at risk from malaria - in 2016, at least 212 million cases were reported, with 445 000 fatalities, most of these being amongst African children (Mandala et al. 2018). Malaria is also developing resistance to current drugs. Co-infection with HIV exacerbates the dangers of malaria (Mandala et al. 2018). Even more serious is the risk of co-infections of HIV, TB and malaria, found to be associated with migrant workers and travel to high-risk countries

### Is antibiotic resistance a symptom of a larger problem with the discovery system itself?

Antibiotic resistance is particularly problematic because industry’s ‘retreat from developing new antibiotics is leading to a loss of expertise in both practical and theoretical aspects of antibiotic biology’ and as ‘industry reassigns or retires its microbiologists’, fewer will be trained in academia, as the knowledge base will take years to rebuild (Nathan 2004:899).

However, if the problem is associated with the global pharmaceutical R&D system itself, then it might be necessary to supplement the system with other approaches. Academic research can sometimes fail to take up innovative opportunities to improve research productivity (Rubin & Callaghan 2019). We argue that a problem of unresponsiveness to important societal research imperatives exists across the global R&D system itself.

As stated previously, this literature is not new (see Cowen 2011; Gordon 2016), and there exists formal theoretical economic models that explain why it is becoming harder to make scientific breakthroughs (see Kortum 1997; Segerstrom 1997). We suggest that if evidence exists of the lack progress to date against antibiotic resistance, then we might be headed for a ‘cliff’, whereby the resistance to learning of microbes exhausts our last defences against them. This evidence points to an impending disaster. If disaster risk reduction is a systematic approach to the identification, assessment and reduction of the risks of disaster, then this article seeks to identify threats associated with the failure of the research system to solve the problem of antimicrobial resistance. By identifying appropriate theory, and what it predicts about improving the effectiveness of antibiotic research, we seek to derive useful insights into how to reduce the risk of research failure.

## Methodology

This article applies a conceptual critical review methodology. In doing so, literature and theory are synthesised in order to develop propositions. These propositions are then used to develop a research agenda. Recommendations are then derived from this process. The strength of this methodological approach is that it provides a synthesis of novel theory and ideas, and develops testable propositions. Further research is then tasked with testing these propositions. Certain micro-foundations of theory are now considered, which might suggest how to mitigate the threat of research failure associated with antibiotic resistance.

## Theoretical micro-foundations

Which theoretical frameworks are useful in reconceptualising scientific research as a real-time process? What are the theoretical mechanisms that predict how this could be performed? Leveraging collective human intelligence is perhaps one approach to address the above questions (Callaghan 2018a).

Micro-foundational theory is a body of the theory that encompasses theoretical relationships that predict how to improve specific aspects of the research process, so as to attain near real-time research productivity. These aspects are fed into the disaster response process, which requires real-time knowledge of how to address the problem of microbial resistance. Macro-foundational theory relates to theoretical predictions about the overarching context of research productivity, or relationships at the aggregate level. Here, we limit our discussions of the disaster response to the research process, which we consider to be particularly important in light of the failure of the research response to the problem of antibiotic resistance to date.

Building on Malone, Laubacher and Dellarocas’s (2009) ideas, Callaghan (2018a) described this body of theory as ‘networked science theory’, drawing on Nielsen’s (2012) term, and Nielsen’s arguments that changes to the discovery process (the scientific research process) itself herald a new age in human development. Indeed, Nielsen (2012) argued that those looking back from the future will identify two periods in human history, namely the pre-networked science and post-networked science eras. For practical examples of how the research process itself is being accelerated through the application of the principles of networked science, see Nielsen (2012).

**FIGURE 1:**
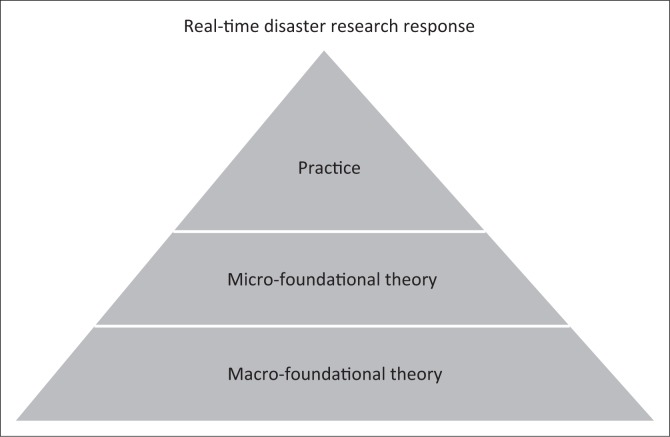
Levels of theory supporting the practice of real-time disaster response.

Nielsen’s (2012) examples offer us useful insights into the practices of research that successfully harness human collective intelligence. We argue here that these examples also offer useful insights into how to attain real-time disaster research response. The practice of networked science, which we envision to provide important guidelines for how to attain real-time research capabilities, or the potential for real-time disaster response, is shown as the top portion of the pyramid in Figure 1. The practice, however, is underpinned by micro-foundational and macro-foundational theoretical frameworks.

Micro-foundational theory relates to the question of how the front line of the research can be pushed back, or how economies of scale can be developed within the research process itself. According to Bernstein, Klein and Malone (2012:41), it is now increasingly possible to solve problems using networks of humans and computers, a phenomenon that has been termed ‘collective intelligence, social computing or various other terms’. Although computer applications and open-source software packages have been used in drug design for many decades, novel theory suggests that such approaches have the potential to contribute to economies of scale in the research process, if used more extensively, and if certain theoretical principles are applied to this purpose. Our argument here is that investments in developing knowledge in this area also suffer from the societal benefit ratio effect – the number of people or resource costs affected by antibiotic resistance (the denominator) is much larger than the numbers of people or resources involved or invested in researching how to address the problem. An important contribution of our argument is its effect as a heuristic to help to focus attention on the problem of very small societal benefit ratios.

Crowds of people can now ‘share, recombine, and refine each other’s creative outputs’ in open idea ecologies, and these approaches have led to successes like youtube.com, Wikipedia.org and Linux (Bernstein et al. 2012:41). Such an approach, although already prevalent in scientific research, might have the potential to radically reduce time and costs in the R&D process, if used more extensively.

Commercial sites like InnoCentive have demonstrated proof of concept, putting scientific problems online for the crowd to solve (Malone et al. 2009). In this way, complex scientific problems are solved more quickly and cheaply than is possible with closed models of in-house R&D. The primary constraint of the InnoCentive model, however, is perhaps the size of the rewards put up for the solving of a particular problem (Callaghan 2017). Radically increasing the size of the reward might increase the incentives of solvers, and the speed at which solutions can be found. Antibiotic resistance is perhaps a useful candidate for this approach. Micro-foundational theory therefore contributes directly to improving the economies of scale that are attainable in the research process itself.

## Theoretical macro-foundations

There are certain insights that have recently emerged from the field of big data analytics. According to Kitchin (2014), the advent of the ‘big data’ era has led to a ‘new epistemological paradigm’ differentiated from traditional deductive ‘knowledge-driven science’. According to Callaghan (2018b), implications of this perspective are not only that interconnectivity and interdisciplinary research will increasingly be enabled by exponentially increased data availability but also that new modes of theory development are now possible.

### Permutation-based science

The primary macro-level theoretical frameworks offered by Romer (1990) and Weitzman (1998) seem to suggest that breakthroughs in R&D might come through the recombinant processes of innovation and ensuring that ideas are shared in the problem-solving process. Given the advent of the big data era, however, it would seem that the ‘problem space’ of science has radically expanded. If problem-solving can be represented as a topography, or a space, then Romer’s and Weitzman’s theoretical frameworks predict that the solutions to R&D problems like antibiotic resistance lie in the ability to theorise and test permutations of presently existing knowledge.

Thus, the first macro-theoretical principle of this article is derived here, that it is only the permutation-based era of science that may hold the promise for achieving economies of scale in the R&D process. The attainment of these economies of scale might be a necessary condition for the solving of pernicious problems like antibiotic resistance. Therefore, we suggest:

*Proposition 1*: The pre-permutation mode of science will experience diminishing returns to R&D investments.

We therefore argue here that the pre-permutation mode of science is simply not able to allocate the required research resources in a way that matches the societal costs of certain problems. This problem is arguably reflected in societal R&D ratios that suggest that vast positive externalities remain untapped in the contemporary global R&D system.

If it is only permutation-based science that can solve problems like antibiotic resistance before its catastrophic consequences, we need to ask what dominant constraints exist to its adoption? What overarching theoretical guidance can we glean from macro-level theory? A consideration of the seminal knowledge aggregation problem might offer us useful insights in this regard.

### The (seminal) knowledge aggregation problem

Probabilistic innovation theory (PIT) (Callaghan 2017) predicts that radically improved cost and time savings can result when problem spaces are ‘populated’ with large numbers of solvers. This theory suggests that by balancing the societal innovation ratio, it is possible to unfold a problem space by populating it with large numbers of people in proportion to the importance (those affected by) of a particular knowledge problem. A real-life example of this can be found in the form of InnoCentive, a site that puts scientific problems online to be solved for reward, using an open call.

Probabilistic innovation theory seeks to reframe the focus of theory development through the use of a heuristic to focus research problem-solving on knowledge aggregation, which is taken to be the dominant constraint for progress in R&D. Drawing from the princsiples of PIT (Callaghan 2016a, 2016b, 2017, 2018a), this can conceptually described as follows:

*ξ* = *f*(time * resources)^*∂*^

This heuristic suggests that the probability of solving any solvable problem (*ξ*), although necessarily a function of complex interactions of time and resources, is primarily subject to the exponential influence of constraints associated with the knowledge aggregation problem (∂). This heuristic accords with predictions of the importance of knowledge aggregation (Hayek 1945; Von Hippel 1994) in knowledge creation, particularly in terms of how to aggregate tacit knowledge (Nonaka 1994), which is at the level of the individual (Polanyi 1973). An implication of this heuristic is that a focus on improving knowledge aggregation might ensure that theory development is targeted on the most important link in the aggregated problem-solving process. On the basis of these logics, the following proposition is derived:

*Proposition 2*: Investments in research that are focussed on improving knowledge aggregation will have substantially higher payoffs than those focussed merely on time and costs in the research process.

It must be noted that the quality of inputs into the Wikipedia-type research process is a concern. An ethical framework premised on the principles of post-normal science (Funtowicz & Ravetz 1994), however, suggests useful principles that can be applied to maximise accountability and transparency in the open research process.

Have certain characteristics of the academic system contributed to a widening societal R&D ratio? From the perspective of computer science, Brookes (1996) issued the following warning over two decades ago:

[…*W*]e tend to forget our users and their real problems, climbing into our ivory towers to dissect tractable abstractions of those problems, abstractions that may have left behind the essence of the real problem. We talk to each other and write for each other in ever more esoteric vocabularies, until our journals become inaccessible even to our society members, and publication properly commands a higher price from the author in page fees than from the reader in subscription fees. (p. 62)

Key to real-time knowledge aggregation is perhaps the leveraging of collective intelligence. Brookes (1996:64) argued that ‘intelligence amplifying systems can, at any given level of available systems technology, beat AI [artificial intelligence] systems’, or in other words, ‘a machine and a mind can beat a mind-imitating machine working by itself’. To obtain economies of scale in the research process, it is necessary to leverage human problem-solving capability, and this is perhaps only possible if the search for opportunities to apply novel technologies to the research process becomes an important stream of research, in itself. In other words, if sufficient researchers populate the numerator of its societal R&D ratio, and if the contributions of these researchers are leveraged using appropriate technologies, then this might turn the tide in certain societally important areas of science, including the development of new antibiotics; hence, the following proposition:

*Proposition 3*: Only through a focus on balancing societal R&D ratios, is it possible to solve most contemporary societally important research problems?

The corollary of this proposition is that problems like antibiotic resistance can only be consistently solved through a radical re-conceptualisation of our education systems and the supply of researchers in the system. Knowledge of the incentives structures within science itself may also require careful scrutiny. If the attractiveness of laboratory-based science is not sufficient to attract the numbers of problem solvers required for a balanced societal R&D ratio, then novel theorising is required urgently.

## Virtualisation of the research or research and development process

As stated above, strengthening the numerator of the societal R&D ratio might occur by simply populating an unfolded research problem space with large numbers of researchers. This can currently be performed using methodologies like crowdsourced R&D. If technology can be used to leverage the contributions of these researchers included in the numerator, then their societal impact might be substantially increased, relative to the denominator, or the numbers of people who bear the consequences of a failure to solve a societally important knowledge problem. This type of approach is not new. According to Brookes (1996:64), more attention should be paid to how humans can work synergistically with artificial intelligence, and computer graphics research may be key to this.

Theory development should therefore be targeted at the potential to leverage, or amplify, collective intelligence (Malone et al. 2009; Nielsen 2012) in the knowledge creation process, but with a specific focus on knowledge aggregation, and targeted attempts to transcend the knowledge aggregation threshold. Research should seek to identify and quantify the dimensions of the knowledge aggregation threshold associated with different societally important research problems (Callaghan 2017).

We suggest here that appropriate processes within the research system should be mapped, and assessed as to their degree of resistance to virtualisation, with a goal of virtualising whichever processes and entire systems that are appropriate in the pursuit of a virtualised discovery system. A virtualised discovery system is a system of research that taps into the near-zero marginal cost model of digital processes. Although the arguments made here are broad, and their granularity is necessarily limited, being at a certain level of abstraction, we suggest that if viewed through the heuristic lens of knowledge aggregation and societal R&D ratio logics, then these insights may provide a useful focus for further theory development. The following proposition is derived from these discussions:

*Proposition 4*: The virtualisation of the research or R&D process is a necessary condition to enable the research productivity required to balance societal R&D ratios.

A corollary of this proposition is that a radical re-conceptualisation of the research process is necessary in order to solve problems like antibiotic resistance. In its current form, it seems as if the probability that it can adequately address threats such as antibiotic resistance is uncertain. These propositions are taken to be necessary conditions for a globally responsive R&D system. It must be acknowledged that the discussions here, and the propositions derived, are framed at a certain level of abstraction. This is arguably necessary, as from a vantage point of theory, it is possible to make predictions about certain patterns of relationships that are less visible at the micro level. A synthesis of the discussions is now undertaken, and recommendations are derived for further research and practice.

## Synthesis and recommendations for theory and practice

**FIGURE 2:**
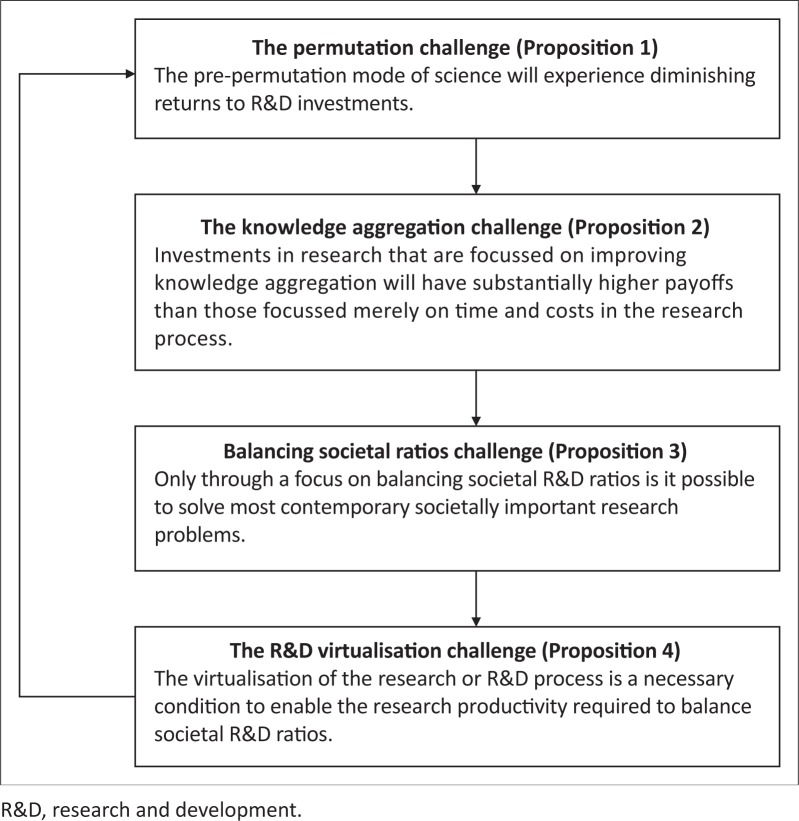
Necessary conditions for solving contemporary societally important research problems.

We argue that the four propositions derived here relate to four challenges faced by the current global R&D system. Figure 2 summarises these four propositions and relates each to one of these four challenges. For a system to offer a more certain or predictable likelihood of solving problems like antibiotic resistance, we argue that these challenges need to become the focus of further theorising and theory development. The first challenge summarised by Proposition 1 relates to the need to complement current research processes with methods that can unfold both problems and the problem research landscapes, or problemscapes. Such methods are perhaps appropriate to an era of permutation science, where radically increased effort is required to make progress under conditions where it is increasingly difficult to develop new knowledge (Gordon 2016; Kortum 1997; Segerstrom 1997). Examples of the decomposition of complex problems (Nielsen 2012) suggest that this is a promising area of focus, in that if such problems can be modularised, then they can be solved by open systems like crowdsourced R&D (Callaghan 2016a). Similarly, if systems and processes apply novel technological developments to the research process, over time it will become easier to unfold the problem space by enabling large numbers of appropriately matched problem solvers to populate the problem space.

While Proposition 1 relates to the challenges of surmounting a pre-permutation mode of science, Proposition 2 relates to the challenge of finding a theoretical framework that can focus further theorising and theory testing. An appropriate theoretical framework might focus research effort and reduce wasted or redundant work that is iterative and is not explicitly linked to a common theoretical frame. If this challenge can be overcome, then this body of literature might provide increasingly important insights over time. A focus on knowledge aggregation might, in turn, focus research efforts on the ultimate outcome of near real-time research productivity. Such research might be particularly important under conditions of disaster management, where the data required for the solving of a knowledge problem only become available after the onset of the disaster. The ultimate challenge of knowledge aggregation theory is to offer useful insights and principles that are effective under such conditions, where the time dimension of research needs to be ‘crashed’. Importantly, addressing the knowledge aggregation problem entails a focus on collective problem-solving, and how to leverage this collective intelligence in real time.

Another important implication of a focus on the knowledge aggregation problem is that it is well suited to global connectivity, and it provides a useful umbrella concept to link research seeking real-time research with a growing body of research that is exploring the potentials of human problem-solving in contexts of heightened connectivity. A focus on near real-time research productivity is arguably useful, in that we might have become inured to a plethora of problems that may have created a tolerance for slow research problem-solving. Why do we need to live with problems like antibiotic resistance, cancer, diabetes, global warming and global conflict? More inclusive theoretical frameworks that are premised on near real-time collective problem-solving might have much to offer in time to come as technology makes such a vision possible.

Proposition 3 extends a consideration of the previous two challenges into methodological terrain, suggesting a benchmark that needs to be achieved before the outcomes of the research process can become ‘probabilistic’ or less uncertain. According to PIT, it is necessary to balance the inputs of the R&D process to the consequences of not solving a problem. If millions stand to be directly affected by rising antibiotic resistance, then, to take this theoretical prediction to the extreme, by having millions of appropriate problem-solvers to populate the numerator of the societal R&D ratio, this balance can be achieved. This is a radical notion, particularly for those accustomed to a global biomedical laboratory-based research system that has to date delivered solutions to many of our biomedical problems. Probabilistic innovation theory suggests that a change in mindset is necessary, and that only by balancing societal R&D ratios can we solve problems like antibiotic resistance in a sustainable way. Addressing this challenge might challenge our acceptance of ailments such as diabetes, cancer and increasing health problems associated with ageing. Given the ageing populations in certain regions of the world, such theory will be expected to increase in its importance over time.

The R&D virtualisation challenge that proposition 4 gives rise to relate to the need to change the mindsets of those who are responsible for applying novel developments in technology to the research process itself. This challenge is perhaps as much a conceptual challenge relating to the need to develop a vision to de-link appropriate aspects and processes of the R&D system from unnecessary constraints associated with bricks and mortar models of these processes. The relatively extensive review of literature relating to antibiotic resistance undertaken in the first half of this article was perhaps necessary, in order to frame the subsequent discussions. If antibiotic resistance were to be treated in the same way that many treat heart disease, diabetes, cancer and most of the ailments associated with ageing, we suggest that a return to a pre-antibiotic age might soon no longer be a possibility but a probable outcome. Hence, we suggest the propositions here as heuristics to provoke thinking and to spur action so as to avoid such a fate.

## Conclusion

The objective of this article was twofold. Firstly, it sought to make an argument that certain knowledge problems exist that are currently not being adequately solved by what was described as the current global R&D system. In doing so, literature relating to the problem of antibiotic resistance was reviewed. Secondly, by developing heuristic principles at a certain level of theoretical abstraction, the article sought to offer certain propositions to guide further theory development related to how to improve R&D systems to solve societally important problems like antibiotic resistance.

Four propositions were derived from literature and theory. Each of these was related to challenges facing R&D productivity. The first challenge was identified as the need to complement R&D systems to be able to accommodate what was termed a permutation-based era in science. The second challenge was argued to relate to how a focus on knowledge aggregation for further theory development may save time and research effort through a focus on leveraging collective human problem-solving capabilities, which can now be enabled by new technologies in a way that was hitherto not possible. The third challenge relates to the need to quantify the productivity of the global R&D in societally important terms, and to balance the societal R&D ratio, or the number of people on the front line of scientific problem-solving of a specific scientific problem as the numerator and the number of people directly affected by the consequences of not solving this same problem as the denominator. The fourth challenge relates to the need for systems and processes in the global R&D system to become radically more responsive to the opportunities offered by novel technologies.

Further research might build on the propositions developed here, and empirical testing might offer useful insights into which of these theoretical propositions generalise across contexts, and under which boundary conditions they do not. We suggest that problems like antibiotic resistance require urgent theory development, lest the innovativeness of microorganisms turns out to be more effective than that of our R&D systems. The following practical recommendations were derived from these conclusions:

- Disaster risk researchers should immediately consider how to embrace the permutation mode of science, and find ways to apply the latest technologies to improve the way they do science. Being aware of new technologies and proactively looking for ways to apply them to improve one’s research productivity may be a useful way to speed up the research process without compromising quality and rigour. The nature of disaster management makes saving time in the knowledge creation process particularly important. The ideas presented here may be particularly useful for disaster management research that needs to occur after a disaster has occurred, and data regarding this process are only available after its onset.
- Disaster risk researchers should proactively strive to improve knowledge aggregation in their research – to also include large-scale collaborations and multidisciplinary collaborations in their research strategies. They should seek to leverage their specialist knowledge by working with others who have complementary skills and knowledge. Real-time research will ultimately require stronger networks between researchers. Disaster management requires multidisciplinary collaborations, and the insights offered here might be particularly useful in that they include a focus on improving the responsiveness of the knowledge management process, incorporating consideration of both multidisciplinarity and real-time sensibilities in its implications.
- Disaster risk researchers should proactively seek to ensure balanced societal benefit ratios, and to develop an awareness of imbalances between the societal costs of problems and societal investments in solving the same problem. Problems associated with climate change, for example, may require more investment, commensurate with its potential for global harm. Disaster risk might be reduced through the use of societal benefit ratios, as useful heuristics in decision-making and resource allocation.
- Disaster risk researchers should look to the potential benefits of virtualising aspects of their research process. Virtualisation might be particularly useful for ideation, and for overcoming physical or geographical limitations to collaborations. Real-time research production may require extensive virtual engagement amongst collaborators.

The ‘takeaway’ of this article is its identification of evidence that suggests that there are theoretical reasons for why research is failing to solve problems like antibiotic resistance, a useful example of what might result in a catastrophic disaster. Its contribution lies in how it synthesises other literature, deriving novel theoretical predications that demonstrate how this failure to avert an impending disaster might be addressed. Although we do not claim to have developed solutions to the wicked problem of the failure of research to solve important societal problems, we do suggest that we have identified certain useful ideas that further disaster risk research can build on. We also suggest that time is of the essence, and that the longer it takes to develop real-time research response capability to respond to disasters, the greater will be the societal costs.
